# Palaearctic leaf beetle *Chrysolinafastuosa* (Coleoptera, Chrysomelidae, Chrysomelinae) new to North America

**DOI:** 10.3897/BDJ.11.e103261

**Published:** 2023-07-11

**Authors:** Hume B Douglas, Tyler W Smith, Patrice Bouchard

**Affiliations:** 1 Agriculture and Agri-Food Canada, Ottawa, Canada Agriculture and Agri-Food Canada Ottawa Canada; 2 Botany, Agriculture and Agri-Food Canada, Ottawa, ON, K1A 0C6, Canada, Ottawa, Canada Botany, Agriculture and Agri-Food Canada, Ottawa, ON, K1A 0C6, Canada Ottawa Canada

**Keywords:** invasive alien species, adventive species, biological control, weed biology

## Abstract

**Background:**

The univoltine leaf beetle *Chrysolinafastuosa* (Scopoli, 1763) is native to in the Palearctic Region from eastern Siberia to western Europe.

**New information:**

First North American records are presented for *C.fastuosa* (Scopoli, 1763) (Coleoptera, Chrysomelidae, Chrysomelinae), as confirmed by vouchered specimens from Canada: Nova Scotia. Additional citizen science records from USA: Vermont are also discussed. Diagnostic information is presented to distinguish *C.fastuosa* from other North American Chrysomelidae and a species distribution model to assess its potential spread in North America is presented. This insect is expected to cause some feeding damage to above-ground parts of ornamental and invasive Lamiaceae, especially species of *Galeopsis* L. The species distribution model and the range of its host plant *Galeopsistetrahit*, suggest the north-eastern US and south-eastern Canada, from the Atlantic coast to the west end of Lake Superior provide the most suitable conditions for this species. The United States of America and Canada are now known to be home to 70 or more species of adventive Chrysomelidae.

## Introduction

The univoltine leaf beetle *Chrysolinafastuosa* (Scopoli, 1763) is native to the Palearctic Region from eastern Siberia to western Europe (Kippenberg 2010). It is a widespread and common species in some countries (du Chatenet 2002, Rheinheimer and Hassler 2018), feeding on several weedy Palaearctic genera of Lamiaceae, including *Galeopsis* L., *Lamium* L., *Leonurus* L., *Prunella* L. (Garin et al. 1999) and also *Urtica* L. (Rheinheimer and Hassler 2018). *Chrysolinafastuosa* is known to complete its life cycle on *Galeopsis* spp. and occasionally *Lamium* spp. in open and semi-shaded habitats (Rheinheimer and Hassler 2018). We first became aware of citizen-science images of *Chrysolinafastuosa* in Nova Scotia (NS) Canada and Vermont (VT) USA, North America on the internet platform iNaturalist (iNaturalist contributors and iNaturalist 2023) thanks to coleopterist and iNaturalist user Boris Buche (Berlin), who alerted HD about citizen science records of this species in North America. We aimed to investigate these reports of *Chrysolinafastuosa* in North America and to complete a preliminary assessment of its potential spread using a Maxent species distribution model.

## Materials and methods

Agriculture and Agri-Food Canada researchers PB, B Brunet and J Gleason conducted field investigations to investigate citizen science records of *C.fastuosa*. They searched for *C.fastuosa* adults on Lamiaceae for about three hours while visiting central Nova Scotia during 2022. We also contacted Nova Scotia entomologists and Dr. Donald Chandler, Emeritus Curator of the University of New Hampshire Insect Collection to search collections for additional specimens (none found). PB collected two adult specimens in Dartmouth NS within 20 m of an individual first reported by iNaturalist contributors earlier in 2022 (https://www.inaturalist.org/observations/122742243).

HD reviewed iNaturalist records of *C.fastuosa* in North America for verification. He also reviewed images of the similarly metallic *Chrysochusauratus* (Fabricius, 1775) (Chrysomelinae, Eumolpinae) from north-eastern North America to search for additional records. Except for the shorter lobes of tarsomere 4 in *Chrysolina* spp., most subfamily-level diagnostic characters (Riley et al. 2002) were not visible in dorsal habitus photos (diagnostic information below). Specimen occurrences were mapped using SimpleMappr (Shorthouse 2010).

As a preliminary assessment of the potential distribution of *C.fastuosa* in North America, TWS prepared a species distribution model using the program Maxent version 3.4.4 (Phillips et al. 2017). All "research grade" iNaturalist records from Europe and Asia were downloaded from GBIF.org (2023a). Records were thinned to one observation per 10 minute grid cell, matching the resolution of the environmental variables (see below). Following thinning, 1021 observations were retained for model training. In addition, we added a point (the country centroid) for every country in which *C.fastuosa* is listed in Kippenberg (2010) for which there were no records in iNaturalist (n = 8: Afghanistan, Albania, Georgia, Greece, Kazakhstan, Liechtenstein, Macedonia, Turkey).

WorldClim bioclimatic variables (version 2.1, 10 minute resolution; Fick and Hijmans (2017)) served as the environmental rasters for the Maxent analysis. These variables provide biologically meaningful summaries of temperature and precipitation data. Maxent is robust to collinearity amongst variables and differences in correlation structure in training and projection regions can reduce model transferability (Feng et al. 2019); accordingly, we used all 19 bioclim variables. We defined the background extent for model training as the set of Koppen-Geiger Climatic Zones (Kriticos et al. 2011) in which there was at least one record of *C.fastuosa*. We used the R package ENMeval (Muscarella et al. 2014) to tune model parameters, comparing regularisation values 1-3 and considering all possible feature classes (linear, quadratic, hinge, polynomial and threshold). Models were evaluated with 4-fold cross-validation using the hierarchical checkerboard option of ENMEval and the optimal parameters were selected using the information criteria approach of Warren and Seifert (2011). The default Maxent CLOGLOG output, which ranges from 0-1, was used to interpret projections, with values above the median (i.e. the 50^th^ percentile) considered to be highly suitable, values between the 5^th^ and 50^th^ percentile as moderately suitable and values between the 1^st^ and 5^th^ percentile as low suitability.

We also downloaded records for the host plant *Galeopsistetrahit* L. from GBIF.org (2023b) in order to compare the current distribution of the host plant to the potential distribution of *C.fastuosa*. Analyses and mapping were conducted in R version 4.2.3 (R Core Team 2023), with the packages terra (Hijmans 2023), dismo (Hijmans et al. 2022) and sf (Pebesma 2018). Code and data necessary to reproduce this analysis are included in Suppl. material 1.

## Taxon treatments

### Chrysolina fastuosa

(Scopoli, 1763)

F8CEC09A-3DB3-5C00-9A44-69C4401CE327

#### Diagnosis

**Figure 1.**
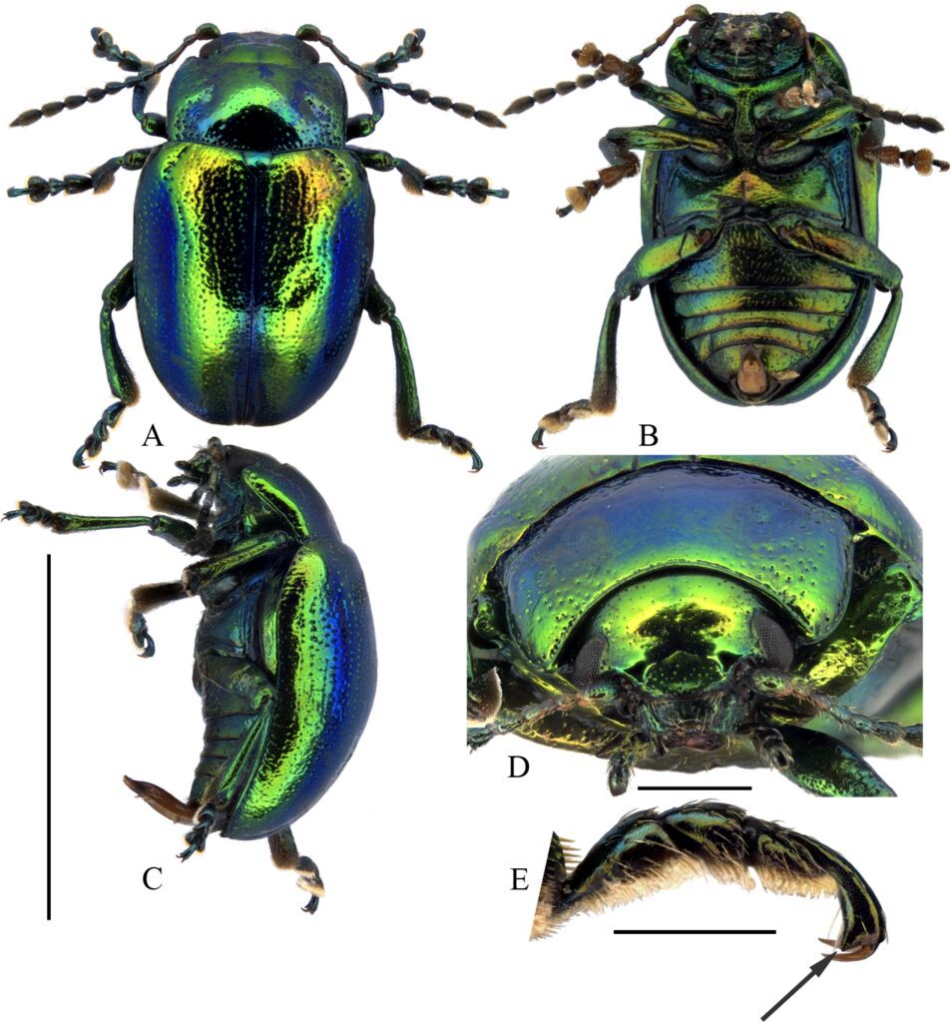
Morphology of a male of *Chrysolinafastuosa* from Nova Scotia, Canada. **A** dorsal habitus. **B** ventral habitus. **C** lateral habitus. **D** anterior view of head. **E** lateral view of protarsomere showing ventro-apical projection (arrow). Scale bars: 5 mm (A-D); 0.5 mm (D, E). Images, K. Savard, AAFC.

*Chrysolinafastuosa* is 5.0-6.0 mm long and can be recognised in Canada and USA as belonging to genus *Chrysolina* by its elongate apical maxillary palpomere, non-connate tarsal claws and metallic elytral colouration (Riley et al. 2002). It can be distinguished from other North American *Chrysolina* by its acute (Fig. 1, E) ventro-apical projection of protarsomere 5 (Rheinheimer and Hassler 2018 –misinterpreted as bidentate tarsal claws, Wilcox 1972). The apex of protarsomere 5 is uniformly narrowed in all other species with no projection. It is also distinguished by the non-concave anterior edges of the compound eyes. It has a brighter metallic green colouration than perhaps any native North American Chrysomelinae, blue metallic at the elytral suture and, in many, also a median longitudinal orange metallic area on the basal 2/3 of each elytron. The truncate aedeagal apex (Fig. 3) is somewhat like *C.inornata* (Rogers, 1856), but distinguished by its median projection.

Individuals of *C.fastuosa* can be distinguished from the similar-looking eumolpine, *C.auratus* by their non-flared metatibial apices without projections. *Chrysochusauratus* is also larger (6.5-10.0 mm) and also has a raised bead at the posterior edge of the prothorax that is greater than 2/3 the width of the base of antennomere 2. In *C.fastuosa*, any visible bead is less than half as wide as the base of antennomere 2. In *C.fastuosa*, antennomere 3 is longer than antennomere 4, while antennomere 4 is longer in *C.auratus*.

#### Distribution

**Figure 2.**
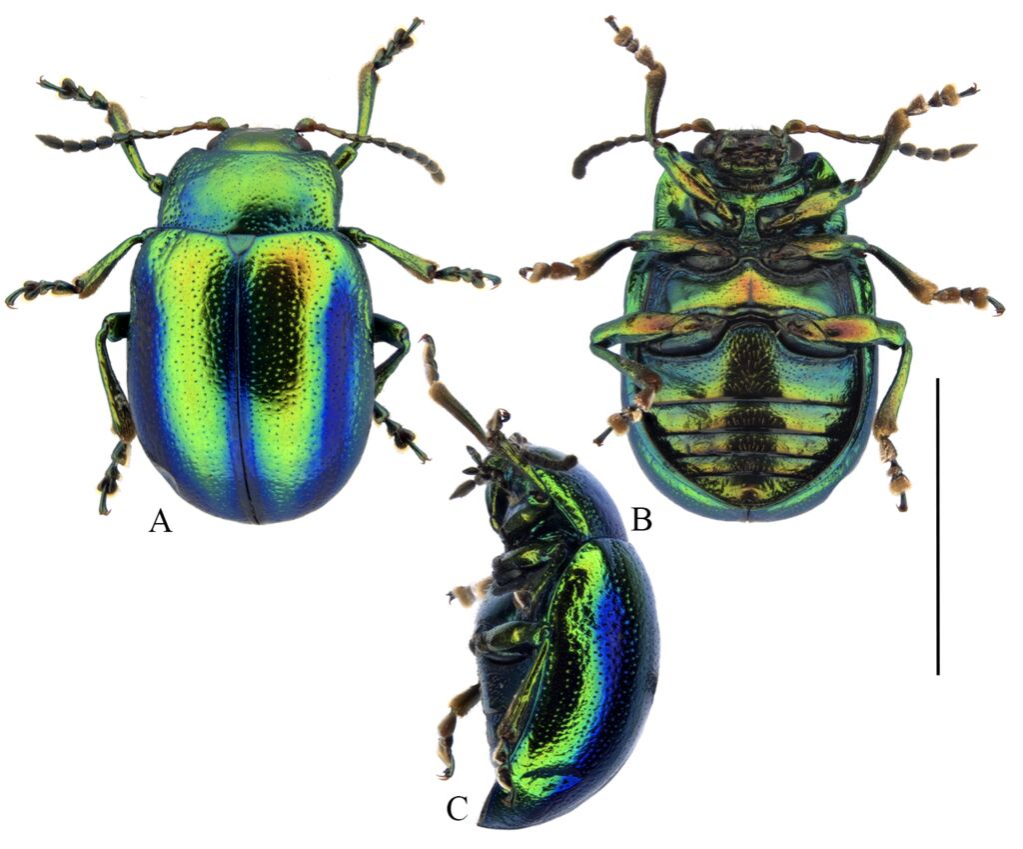
Habiti of a female of *Chrysolinafastuosa* from Nova Scotia, Canada. **A** dorsal. **B** ventral. **C** lateral. Scale bar: 5 mm. Images, K. Savard, AAFC.

**Figure 3.**
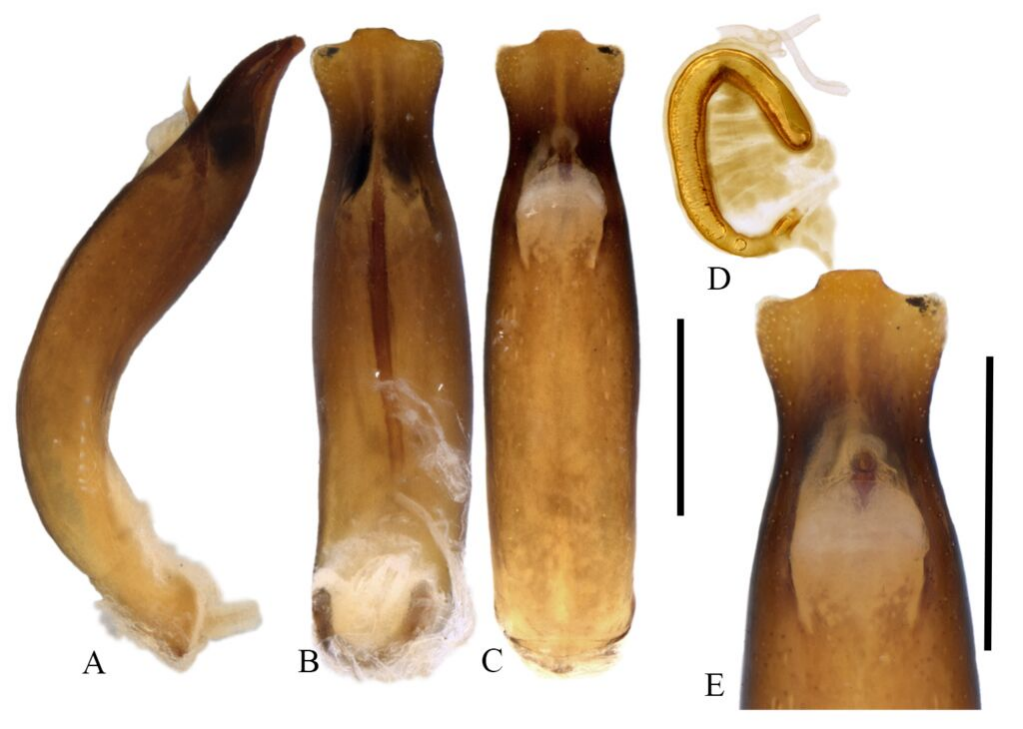
Genitalia of *Chrysolinafastuosa* from Nova Scotia, Canada. **A** Male aedeagus, lateral view. **B** Aedeagus, dorsal view. **C** Aedeagus, ventral view. **D** Female spermatheca. **E** Aedeagus, apex in ventral view. Scale bars: 0.5 mm. Images, K. Savard, AAFC.

PB collected two specimens of *C.fastuosa* (Figs 1, 2, 3) at the following locality in **Canada: Nova scotia**: Dartmouth, Lawnsdale Drive Park Trail 44.683, -63.570. Both are deposited at the CNCI (2023), with database Specimen ID numbers CNC1989589 and CNC1989590. Forty-seven additional individuals were seen in 43 citizen-science observations from Nova Scotia, Canada and Vermont, USA (iNaturalist contributors and iNaturalist 2023, Simpson et al. 2023). These photos were each identified by iNaturalist contributors as being of *C.fastuosa* and were confirmed by HD to match *C.fastuosa* and no other North American chrysomelid species. We present specimen and photographic evidence of 64 individuals from multiple sites in Nova Scotia Canada and Vermont USA, separated by over 900 km over 11 years. These led us to conclude that at least two populations of *C.fastuosa* are established in North America. We expect that these are the only two large populations in North America because this is a conspicuous, day-active species that is apparently readily documented by iNaturalist users.

**Figure 4.**
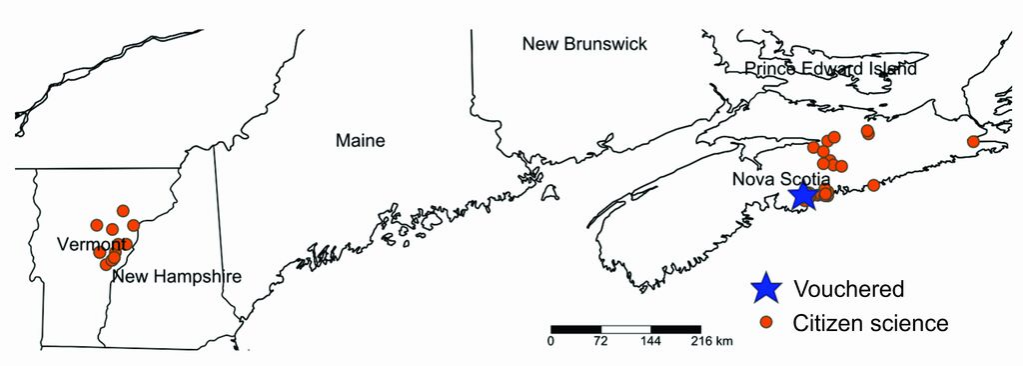
Map of vouchered record and citizen-science records of *Chrysolinafastuosa* from eastern Canada and USA.

*Chrysolinafastuosa* is known from the following localities in North America (Fig. 4). **Canada: Nova Scotia.** Antigonish County: 45.623 -61.993. Colchester County: Truro, 45.358 -63.263; Coldstream, 45.070 -63.317; Colchester, 45.225 -63.317; Wittenburg, 45.110 -63.233. Guysborough County, Guysborough, 45.350 -61.406. Halifax County: Brookvale, 45.037 -63.085; Dartmouth, 44.647 -63.536; 44.675 -63.484; 44.705 -63.532; 44.664 -63.544; 44.680 -63.562; 44.695 -63.561; 44.695 -63.561; 44.699 -63.561; Halifax, 44.59 -63.553; 44.796 -62.676; 44.643 -63.598; -44.667 -63.563; -44.689 -63.501; -44.691 -63.422; -44.800 -63.46; 44.604 -63.517; 44.636 -63.5938; Lawrencetown, 44.669 -63.391; Porters Lake, 44.748 -63.298; Seaforth, 44.663 -63.296; 44.663 -63.296; 44.664 -63.296. Pictou County: Pictou, -45.450 -62.743; White Hill, 45.488 -62.757; 45.488 -62.758. **USA: Vermont.** Caledonia County: East Hardwick, 44.468 -72.238. Orange County: Bradford, 44.044 -72.197; 44.044 -72.197; Strafford 43.843 -72.374; 43.841 -72.381; USA 43.873 -72.350; Strafford, 43.875 -72.351; Vershire, 43.940 -72.337; 43.941 -72.337. Washington County: Plainfield, 44.233 -72.374; -44.286 -72.573. Windsor County: Sharon, 43.785 -72.454.

#### Notes

The external morphology and male genitalia of the Canadian specimens closely matched taxon concepts of *C.fastuosa*. Specimens were confirmed as *C.fastuosa* using Mohr (1966) and Rheinheimer and Hassler (2018) and in comparison with specimens from the Canadian National Collection of Insects Arachnids and Nematodes (CNCI) identified by M. Daccordi (Italy), J.C. Bourdonné (France) and R. de Ruette (Ottawa). As the only member of the subgenus Fastuolina Warchalowski, 1991 (Kippenberg 2010), *C.fastuosa* is unlikely to be confused with any other species.

## Analysis

### Potential Distribution

**Figure 5.**
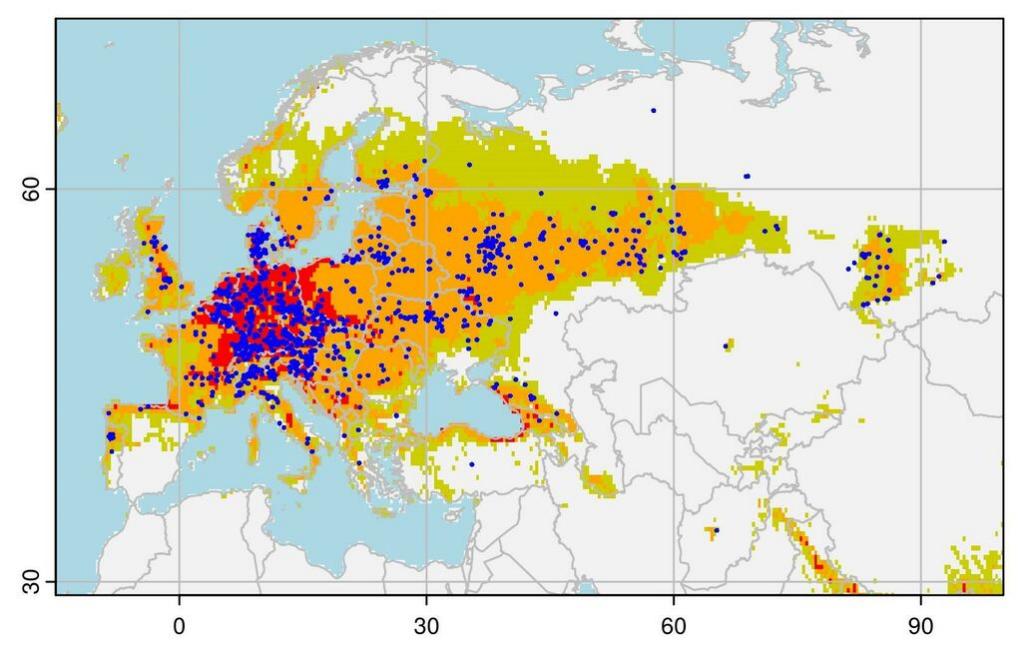
Distribution of *Chrysolinafastuosa* in its native range. Points show iNaturalist records retrieved from GBIF. Shading indicates Maxent suitability models: dark/red areas are the highest suitability (50^th^ percentile and above, CLOGLOG > 0.73), medium/orange areas are moderate suitability (5^th^ percentile, 0.30 < CLOGLOG < 0.73) and light/olive areas are low suitability (1^st^ percentile, 0.08 < CLOGLOG < 0.30).

The optimal Maxent model parameters were regularisation = 1, with all feature classes retained; the mean AUC for the four calibration replicates of this model was 0.900, with a mean difference from the corresponding validation sets of 0.023. The suitability maps closely mirrored the distribution of *C.fastuosa* in its native range, with the highest suitability reported from central Europe, declining eastwards into Russia and northwards into Scandinavia (Fig. 5). The variables with the highest contribution to the model were annual temperature range (bio7, 32.8% relative contribution) and annual mean temperature (bio1, 16.9% relative contribution). The response curves for these variables suggest the optimal annual temperature range for *C.fastuosa* is between 20°C and 30°C, with suitability declining sharply with an annual range below 20°C or above 50°C. Suitability declines when annual mean temperature is below 3°C.

**Figure 6.**
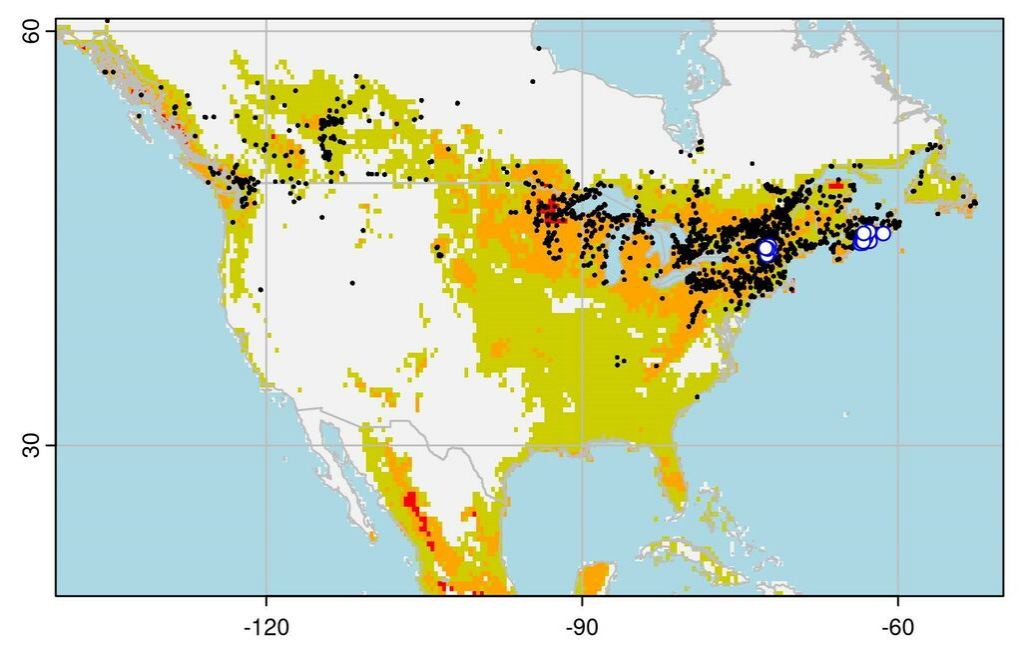
Maxent suitability map for *Chrysolinafastuosa* in North America. The white circles show known occurrences of *C.fastuosa*. Black dots show GBIF records of the host plant *Galeopsistetrahit*. Shading indicates Maxent suitability models: dark/red areas are the highest suitability, medium/orange areas are moderate suitability and light/olive areas are low suitability, with the same thresholds as in Figure 5.

Projecting this model to North America, the new records in Vermont and Nova Scotia fall in regions of moderate climate suitability (Fig. 6). Most of the north-eastern US and the Great Lakes region is classified as moderately suitable, with suitability declining towards the south and north west. The primary host plant for *C.fastuosa*, *Galeopsistetrahit* L. (see below) is most abundant in the northern USA and Canada.

## Discussion

### Adventive Species Biology

Adult *Chrysolinafastuosa* are known to feed on leaves of *Galeopsis* spp., *Lamium* (spp.) and *Urtica* spp in their native range. Larvae are thought to specialise on leaves and floral parts of *Galeopsis* spp. (especially *G.tetrahit*) and perhaps also on *Lamiumalbum* L. and *L.maculatum* L. All species of *Galeopsis* and *Lamium* are of Eurasian origin and not native to North America (Kartesz and BONAP –The Biota of North America Program 2015); *Galeopsistetrahit* is weedy in ornamental and agricultural settings and can cause serious yield reductions in canola, wheat, oats and alfalfa (O'Donovan and Sharma 1987). However, North America is home to three native species of *Urtica* L. (Lamiaceae), including the common and widespread Urticadioicassp.gracilis (Aiton) Selander (Boufford 2023). *Chrysolinafastuosa* uses plants in this genus for adult feeding (Rheinheimer and Hassler 2018). *Chrysolinafastuosa* could possibly harm ornamental ground cover plantings of *Lamium*. However, such feeding may be partly beneficial because *Lamium* species also invade crop fields and native habitats (Swearingen and Bargeron 2016). *Lamiumamplexicaule* L. and *L.purpureum* L. are further agriculturally harmful as winter annual weeds of crop fields on which the soybean cyst nematode, *Heteroderaglycines* Ichinohe, 1952 (Tylenchida, Heteroderidae), can complete development (Creech et al. 2007). Thus, *C.fastuosa* presents both potential beneficial impacts on introduced weeds and an ecological threat to native plant species. More detailed study will be needed before the magnitude of these potential impacts is understood.

*Chrysolinafastuosa* is attacked by several parasitoids in Europe, but may have few predators due to the presence of cardiac glycosides in its tissues (Rheinheimer and Hassler 2018). All known hymenopteran parasitoids are egg parasitoids from the family Mymaridae. Of these, only *Anaphesluna* (Girault, 1914) (= *A.brachygaster* Debauche, 1948) is known from North America (Huber and Thuroczy 2018). However, species concepts in this genus of Mymaridae require further testing (Huber and Thuroczy 2018). The remaining known parasitoids are all from Diptera, Tachinidae, but none of these is known from North America (O’Hara et al. 2020).

The distribution of *C.fastuosa* matches prior observations (Douglas et al. 2021) that most of the accidentally established non-indigenous Chrysomelidae in North America are European weed-associated species that have established in eastern Canada and north-eastern USA. It is possible that *C.fastuosa* was also accidentally first introduced into North America with woody ornamental plants with soil during 1960 to 1965 as hypothesised by Douglas et al. (2021) for several other weed-associated European Chrysomelidae. If this is true, then North American populations have been present for more than 50 years and their expansion has been gradual. Under this scenario, we can expect continued gradual population expansion outwards from central Vermont and central Nova Scotia unless further human-mediated transportation occurs. Such slow spread is consistent with the biology of *C.fastuosa* because most individuals disperse by walking rather than flying (Rheinheimer and Hassler 2018). If North American populations are more recent, then they may be spreading more quickly.

The distribution model for *C.fastuosa* indicates that the distribution of suitable climatic conditions in North America largely corresponds to the distribution of its host plant *Galeopsistetrahit*: primarily, the area from New Jersey to Nova Scotia, west to Lake Superior. While *G.tetrahit* occurs across the prairies and into British Columbia, climatic conditions in the continental interior may present a barrier to the natural dispersal of *C.fastuosa* through this region. However, while *C.fastuosa* is relatively rarely observed in regions with low climate suitability in its native range (Fig. 5), it is not entirely absent, so we must be cautious in interpreting the distribution limits suggested by our model. The analysis presented here is preliminary and more detailed study, including physiological data, is necessary to produce a more rigorous assessment. Furthermore, our analysis is subject to the limitations of all correlative distribution models: un- or under-reported occurrences and misidentifications in the source data (iNaturalist) may introduce bias. However, we note the records we use correspond closely to the distribution reported in the Palaearctic Catalogue of Coleoptera (Kippenberg 2010), which suggests they are adequate for this analysis.

This new North American record, added to the species counts by Douglas et al. (2021) and Douglas et al. (2022) indicate that Canada and the USA are together known to host between 70 and 80 species of adventive Chrysomelidae. Of these, 53 to 61 adventive species of Chrysomelidae are known from Canada and 57 to 67 are known from the USA. This is the eighth or ninth adventive member of subfamily Chrysomelinae established in Canada and USA, of which four to six were introduced accidentally (Douglas et al. 2021).

### Conclusions

*Chrysolinafastuosa* has been established in North America in Canada: Nova Scotia and USA: Vermont on introduced *Galeopsis* L. (Lamiaceae) plants. Numbers of recorded adventive Chrysomelidae for Canada and America north of Mexico are updated to reflect this finding.

## Supplementary Material

XML Treatment for Chrysolina fastuosa

6FE284D3-44A4-5457-BEAB-C1502ED3D254 10.3897/BDJ.11.e103261.suppl1 8142224 Supplementary material 1 Maxent analysis script Data type zip file, containing occurrence records GBIF, shapefiles and R code for processing the data Brief description The main file is chrys.Rmd, which is an RMarkdown (i.e. plain text) file that will reproduce the distribution model analysis included in this paper. File: oo_843642.zip https://binary.pensoft.net/file/843642 Tyler Smith
